# Winter-spring temperature pattern is closely related to the onset of cambial reactivation in stems of the evergreen conifer *Chamaecyparis pisifera*

**DOI:** 10.1038/s41598-020-70356-9

**Published:** 2020-08-31

**Authors:** Md Hasnat Rahman, Kayo Kudo, Yusuke Yamagishi, Yusuke Nakamura, Satoshi Nakaba, Shahanara Begum, Widyanto Dwi Nugroho, Izumi Arakawa, Peter Kitin, Ryo Funada

**Affiliations:** 1 Faculty of Agriculture, Tokyo University of Agriculture and Technology, Fuchu, Tokyo 183-8509 Japan; 2 Institute of Global Innovation Research, Tokyo University of Agriculture and Technology, Fuchu, Tokyo 183-8538 Japan; 3 Institute of Wood Technology, Akita Prefectural University, Noshiro, Akita 016-0876 Japan; 4 Research Faculty of Agriculture, Hokkaido University, Sapporo, 060-8589 Japan; 5 Faculty of Agriculture, Bangladesh Agricultural University, Mymensingh, 2202 Bangladesh; 6 Faculty of Forestry, Universitas Gadjah Mada, Jalan Agro No. 1 Bulaksumur, Yogyakarta, 55281 Indonesia; 7 Department of Bacteriology, University of Wisconsin, Madison, WI 53706 USA

**Keywords:** Plant sciences, Environmental sciences

## Abstract

Temperature is an important factor for the cambial growth in temperate trees. We investigated the way daily temperatures patterns (maximum, average and minimum) from late winter to early spring affected the timing of cambial reactivation and xylem differentiation in stems of the conifer *Chamaecyparis pisifera*. When the daily temperatures started to increase earlier from late winter to early spring, cambial reactivation occurred earlier. Cambium became active when it achieves the desired accumulated temperature above the threshold (cambial reactivation index; CRI) of 13 °C in 11 days in 2013 whereas 18 days in 2014. This difference in duration required for achieving accumulated temperature can be explained with the variations in the daily temperature patterns in 2013 and 2014. Our formula for calculation of CRI predicted the cambial reactivation in 2015. A hypothetical increase of 1–4 °C to the actual daily maximum temperatures of 2013 and 2014 shifted the timing of cambial reactivation and had different effects on cambial reactivation in the two consecutive years because of variations in the actual daily temperatures patterns. Thus, the specific annual pattern of accumulation of temperature from late winter to early spring is a critical factor in determining the timing of cambial reactivation in trees.

## Introduction

Wood is used as raw material for timber, furniture, pulp and paper, chemicals and fuel^1,2^. On global scale, the biological process of wood formation is important for mitigation of climate change via carbon sequestration. Cambium is the meristematic tissue in trees that is responsible for production of xylem and phloem and thus for radial growth of trees^3^. Cambium in trees from temperate environments undergoes alternating annual cycles/periods of dormancy and activity, controlled by internal and environmental factors ^4–8^.

The timing of cambial reactivation from late winter to early spring influences both the quantity and the quality of wood. Earlier reactivation of cambium might potentially increase the duration of cambial activity in *Picea mariana*^9,10^ and resulted in wider xylem increments in* Picea mariana*^10,11^ and in *Populus sieboldii* × *P. grandidentata*^12^. In addition, the duration of the growing season depends on the cessation of cambial activity^10^. Environmental temperature is closely associated with the radial growth of trees^10,13–22^ which is the result of the active growth of cambium during annual vegetation periods. The active period of cambium continues from a few weeks to several months, according to species and, local climates and growth conditions in trees^23–26^. Moreover, it was previously noticed that the annual radial growth is related with the active period of cambium and rate of xylem production in conifers^27,28^ such as *Pinus densiflora*^29^ and *Picea mariana*^10^. The variations in the annual productivity of trees reflect the adaptability of trees to specific regional environmental conditions. Therefore, it was proposed that an increase in radial growth might be the result of a longer growing season and/or a higher growth intensity^24,30^.

When the air temperature starts to rise from late winter to early spring, dormant cambium becomes active under natural conditions^6, 7^. Moreover, an artificial increase in the temperature of stems during cambial dormancy in winter induced cambial reactivation in several experimental conifers^31–40^, a diffuse-porous hardwood^41^ and a ring-porous hardwood^42^. Thus, an increase in temperature from late winter to early spring has been clearly demonstrated to be a direct trigger for cambial reactivation in temperate trees^6,7^. Recent climate projections predict that global temperatures will rise by 3–6 °C within the next 100 years^43^. Therefore, future global warming will lead to an earlier resumption of cambial activity in spring, resulting in longer growth periods in temperate trees^10,44^. However, forecasting the effects of warming on tree growth remains challenging because of the complexity of both climatic factors and wood formation^10,19,44–49^.

In semi-arid regions with dry winters and springs, the onset of xylem growth is not only depending on the temperature but also both of critical temperature and precipitation above the threshold values^19^. In the case of extreme cold temperate regions and at higher altitude and latitude, the duration of the active season of cambium is short which is dominantly regulated by temperature^10,50^. In addition, in the cool temperate regions, seasonal changes in climatic factors other than temperatures, such as daylight or humidity might influence cambial activity^11,19,27,28,44,51^. Moreover, the relationship between temperature and cambial activity is sometimes influenced by the tree species^52,53^, tree age/cambial age^6,37,54,55^, and tree size^20,24,56^. Internal physiological growth regulators such as plant hormones and storage substances are also regulated by the variation in temperature^6,39,52,57^. Studies on how the changing pattern of daily temperatures interacts with other factors and influence cambial activity and xylem growth are essential to our understanding of tree response to changes in environmental conditions.

Wang^49^ proposed that wood formation is initiated in trees when a certain temperature (in terms of degrees) has accumulated over time. More recently, it has been shown that cambial reactivation occurs when temperatures exceed threshold maximum, average and minimum daily temperatures^12,17,23,37,54,56,58^. Rossi et al.^9^ proposed that calculations of such threshold values might be useful in efforts to evaluate the impact of climate change/changing environmental conditions on cambium phenology in trees. Moreover, using threshold temperatures, we proposed the use of a cambial reactivation index (CRI) for calculations of the timing of cambial reactivation in evergreen conifers^37^ and deciduous hardwoods^12^. This CRI model is based on the hypothesis that the dormant cambium reactivates its activity (cell division and xylem production) after sufficient exposure to temperatures above the threshold critical temperature in the spring^12,37^. In contrast, Delpierre et al.^46^ compared three models such as threshold models, heat-sum models and chilling-influenced heat-sum models for the onset of xylem differentiation and found that chilling-influenced heat-sum models performed better for predicting the onset of xylem differentiation in spring in conifers grown in Northern Hemisphere.

In the present study, we examined how the daily temperatures affected the timing of cambial reactivation and onset of xylem differentiation from late winter to early spring under natural conditions in the evergreen conifer *Chamaecyparis pisifera*. The timing of cambial reactivation was investigated by reference to the accumulation of daily maximum temperatures (in degrees) above a threshold value in 2013 and 2014. Our goal was to establish the CRI value and the threshold maximum temperature for *C. pisifera* trees which can be used to calculate dates of cambial reactivation from temperature profiles. Based on our experimental data from 2013 and 2014, we established a model that was verified on the data collected in 2015. Besides, we performed a simple simulation of the effect of predicted climate change by assuming that the temperature had been higher than the actual recorded daily maximum temperatures in 2013 and 2014. By testing the accuracy and predictions of our model, we aimed to provide a more complete understanding of the future use of the CRI model in calculating annual forest productivity and expected large-scale tree responses to climate change.

## Results

### Effects of temperature on cambial reactivation and xylem differentiation

**Figure 1:**
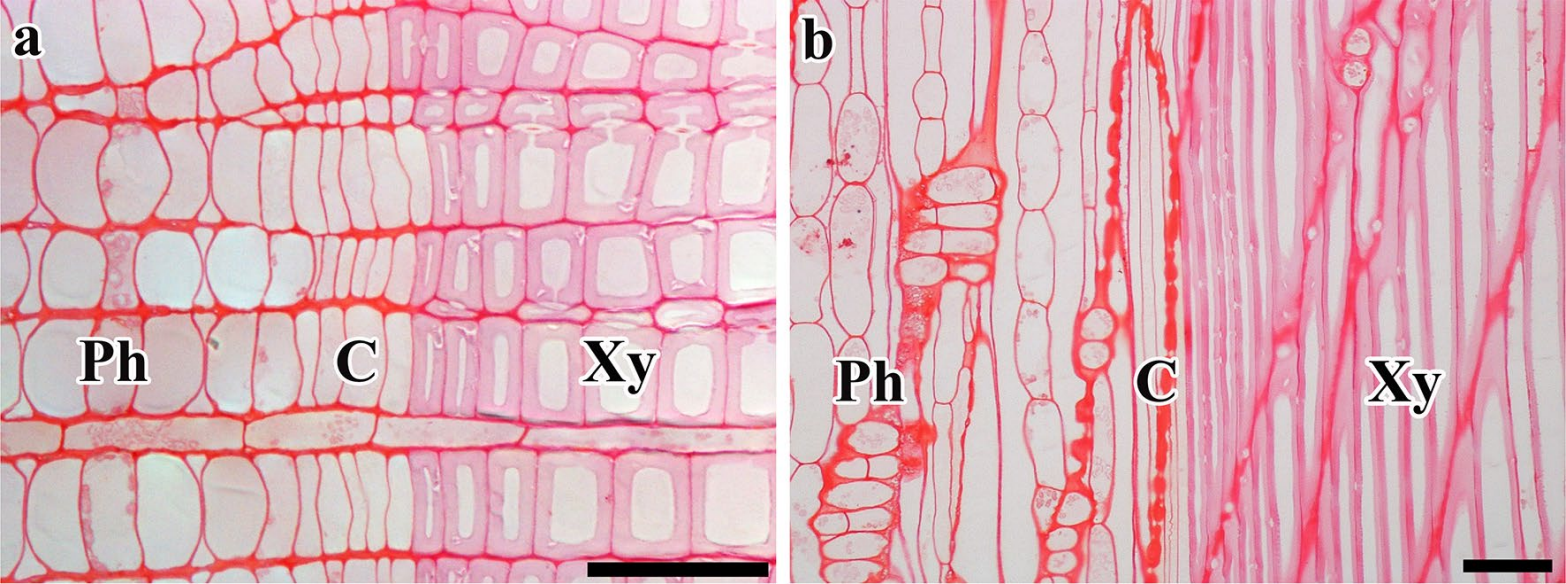
Light micrographs showing the cambium on 28 January 2013. The transverse (**a**) and radial (**b**) views reveal four or five layers of compactly arranged fusiform cambial cells and the absence of new cell plates. *C* Cambium; *Ph* phloem; *Xy* xylem. Bars = 50 µm.

On 28 January 2013 and 2014, samples from two trees revealed three to five compactly arranged layers of fusiform cambial cells in the cambial zone with no evidence of newly formed periclinal cell walls, confirming the cambial dormancy in winter (Fig. 1a,b).

**Figure 2:**
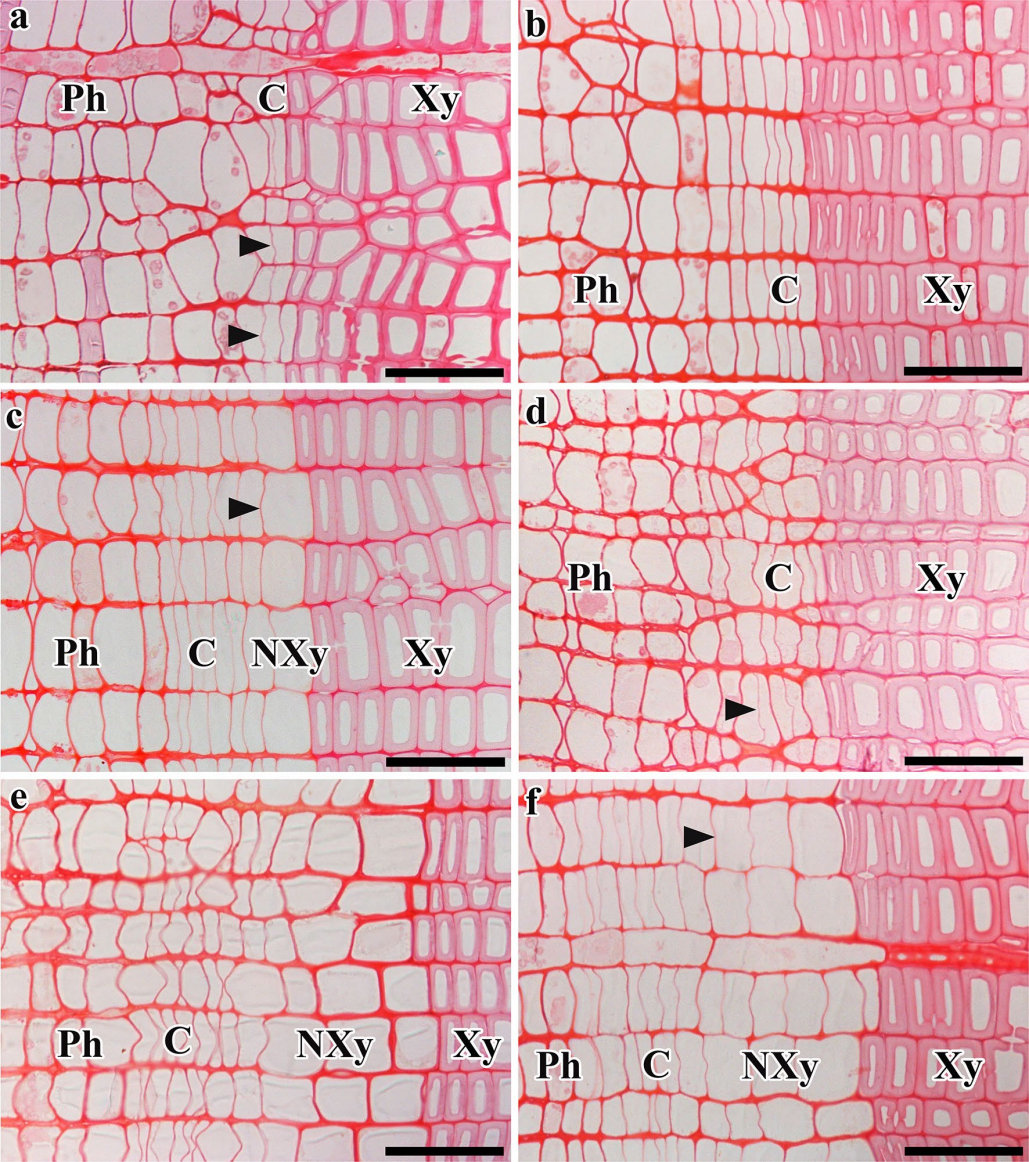
Light micrographs showing transverse views of cambium and its derivatives. In 2013, new cell plates (arrowheads) were detected in the cambium on 10 March (**a**). On the same date in 2014, no new cell plates were visible in the cambium (**b**). In 2013, cambial cells located close to the last year’s latewood were differentiating into tracheids on 25 March (**c**). On the same date in 2014, new cell pates (arrowheads) were detected in the cambium (**d**). On 9 April 2013, two or three layers of earlywood tracheids were detected (**e**). On the same date in 2014, two or three layers of differentiating earlywood tracheids were observed (**f**). *C* Cambium, *Ph* phloem, *Xy* xylem, *Nxy* new xylem. Bars = 50 µm.

The first new periclinal cell walls were found in the cambium of stems of both trees on 10 March 2013 (Fig. 2a). By contrast, on the same date in 2014, the cambium of the same stems was still dormant, with no evidence of periclinal cell wall in the cambium and compactly arranged cambial cells (Fig. 2b). In 2013, on 25 March, the reactivated cambial cells in the cambial zone that were located close to the latewood boundary had begun to differentiate into earlywood tracheids (Fig. 2c). By contrast, in 2014, the periclinal cell walls in the cambium of both stems was observed for the first time on 25 March 2014, indicating cambial reactivation (Fig. 2d). The difference in timing of cambial reactivation between 2013 and 2014 was approximately 15 days.

**Figure 3:**
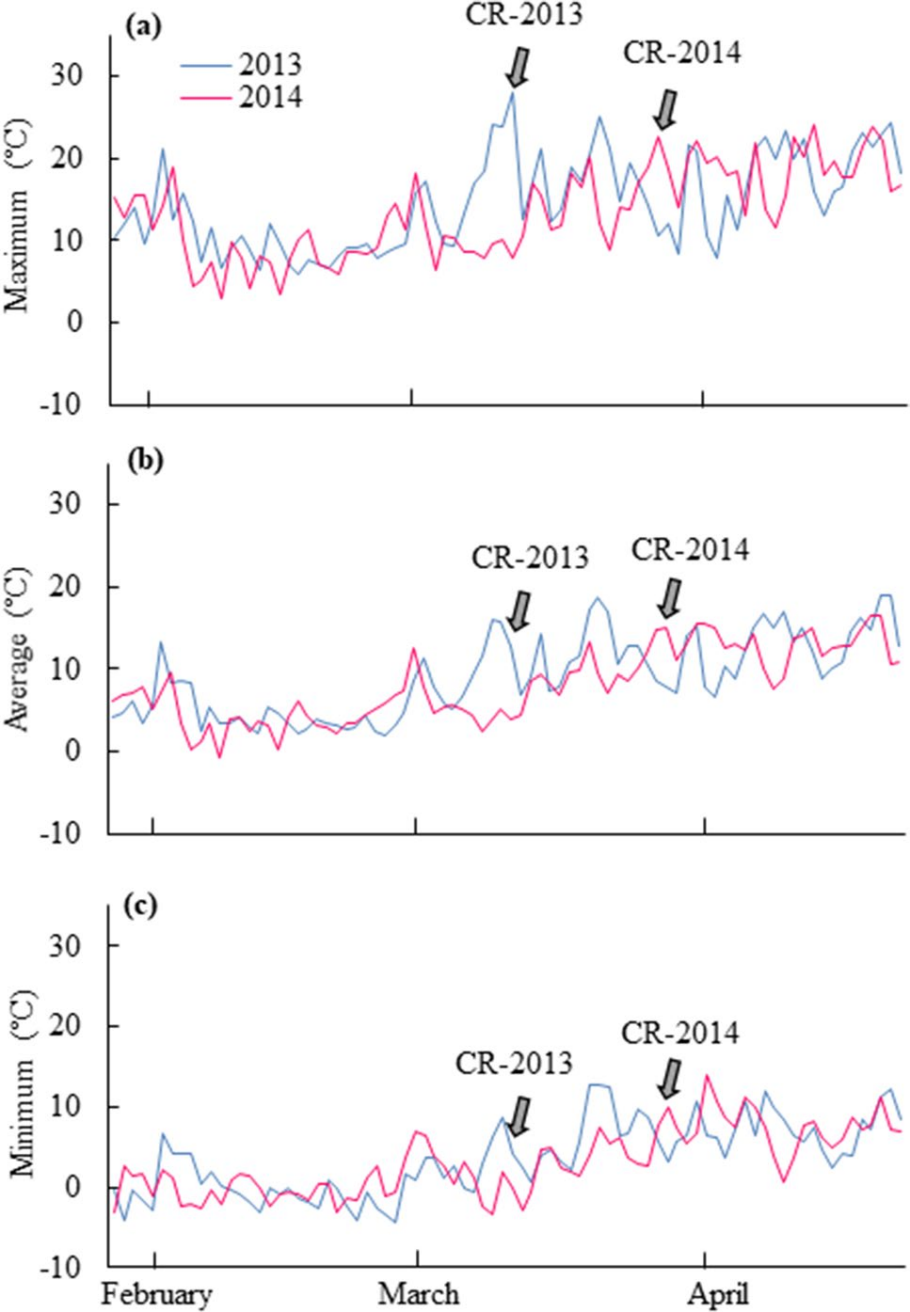
Metrological data showing the maximum (**a**), average (**b**) and minimum (**c**) daily air temperatures at the experimental site in Fuchu, Tokyo, from 28 January to 30 April in 2013 and 2014. Arrows indicate the start of cambial reactivation (CR) in 2013 and 2014.

In 2013, the daily maximum temperature started to increase from around 1 March, rising to a maximum value of 28.1 °C on 10 March 2013 at the time of cambial reactivation. The daily average temperature was 12.9 °C. The lowest value of the daily minimum temperature was above freezing (0 °C). In 2014, on the same date, namely 10 March, the cambium was still dormant and the daily maximum temperature was 8 °C. The average and daily minimum temperatures were 3.9 °C and − 0.1 °C. The daily maximum temperature started to increase from 16 March onwards and reached a maximum value on 25 March (22.6 °C) at the time of cambial reactivation in 2014. On this date, the daily average temperature was 14.8 °C and the daily minimum temperature was above freezing (0 °C). Increases in temperature from late winter to early spring started approximately 16 days earlier in 2013 than in 2014 (Fig. 3a–c).

The onset of xylem differentiation in the cambial zone of both stems began on 25 March 2013 (Fig. 2c). On 9 April 2013, we observed three to four layers of earlywood tracheids (Fig. 2e). In 2014, the formation of earlywood tracheids was initiated on 9 April and three to four layers of earlywood tracheids formed in both stems (Fig. 2f). The difference in the timing of initiation of earlywood tracheids between 2013 and 2014 was 15 days.

The timing of cambial reactivation and formation of earlywood tracheids in the two trees in 2013 and 2014 is summarized in Table 1. In 2013, the first layer of differentiating tracheids in the cambial zone was observed on 25 March, when the daily maximum, average and minimum temperatures were 10.6 °C, 8.6 °C and 5.8 °C, respectively. In 2014, the formation of earlywood tracheids in the cambial zone was initiated on 9 April, when the daily maximum, average and minimum temperatures were 20 °C, 14 °C and 7 °C, respectively, and each was higher than the corresponding temperature in 2013. In each of the experimental years, there were no obvious differences in terms of the date of onset of formation of earlywood tracheids and the progression of cambial activity between the two studied trees.

**Table 1:** The timing of initiation of cambial reactivation and formation of earlywood tracheids in the stems of *Chamaecyparis pisifera* in 2013 and 2014.

Year	Cambial dormancy	Cambial reactivation	Formation of earlywood tracheids
2013	28 January	10 March	25 March
2014	28 January	25 March	9 April

### Analysis and verification of the threshold temperature for cambial reactivation

We calculated CRIs for a range of threshold temperatures (*T*_t_) between 10 and 14 °C, using daily maximum temperatures in 2013 and 2014. When the *T*_t_ was 10 °C, the CRIs were 115 °C and 134 °C in 2013 and 2014, respectively. In 2013 and 2014, CRIs for *T*_t_ values of 11 °C, 12 °C, 13 °C and 14 °C were 96 °C and 106 °C, 79 °C and 84 °C, 66 °C and 65 °C, and 55 °C and 48 °C, respectively. With 13 °C as the threshold temperature, the CRIs of 66 °C and 65 °C for 2013 and 2014 were closer together than those for other values of *T*_t_. Therefore, a daily maximum temperature of 13 °C appeared to be an appropriate threshold value for calculations of the CRI of *C. pisifera* tree stems.

**Figure 4:**
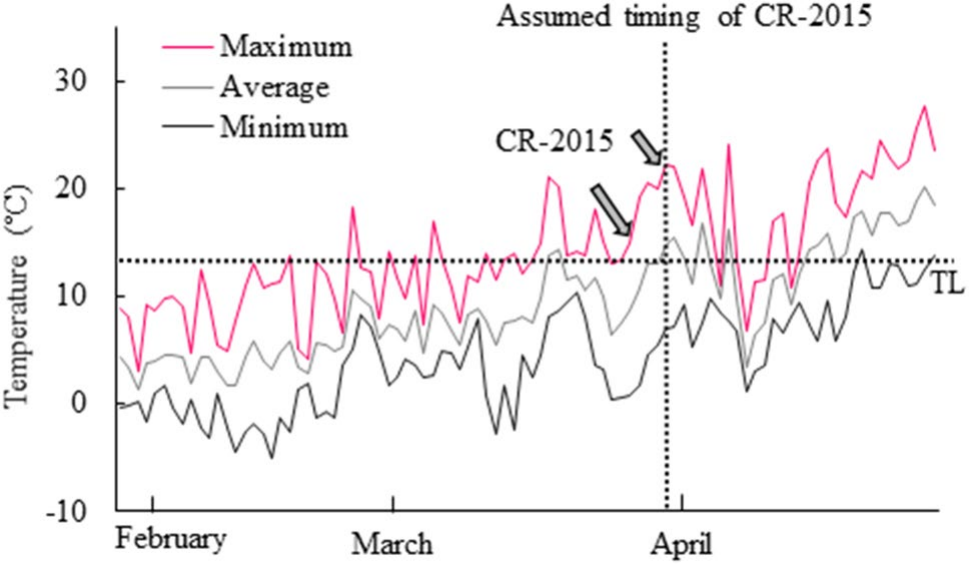
Metrological data showing the daily maximum, average and minimum air temperatures at the experimental site in Fuchu, Tokyo, from 28 January to 30 April in 2015. The horizontal black dotted lines indicate the threshold temperature level (TL), the vertical black dotted lines indicate the assumed date of cambial reactivation (CR) and arrows indicate the actual date of cambial reactivation.

In 2015, we postulated a possible date for cambial reactivation on the basis of the daily maximum temperature profile, from the Japan Meteorological Agency in Fuchu, Tokyo, using the CRI (Fig. 4). The daily maximum air temperatures started to rise consistently from mid-March to the beginning of April in 2015. When we used 13 °C as the threshold temperature, CRI was equal to 65 °C on 29 March. Therefore, 29 March 2015 appeared to be the probable date of cambial reactivation.

**Figure 5:**
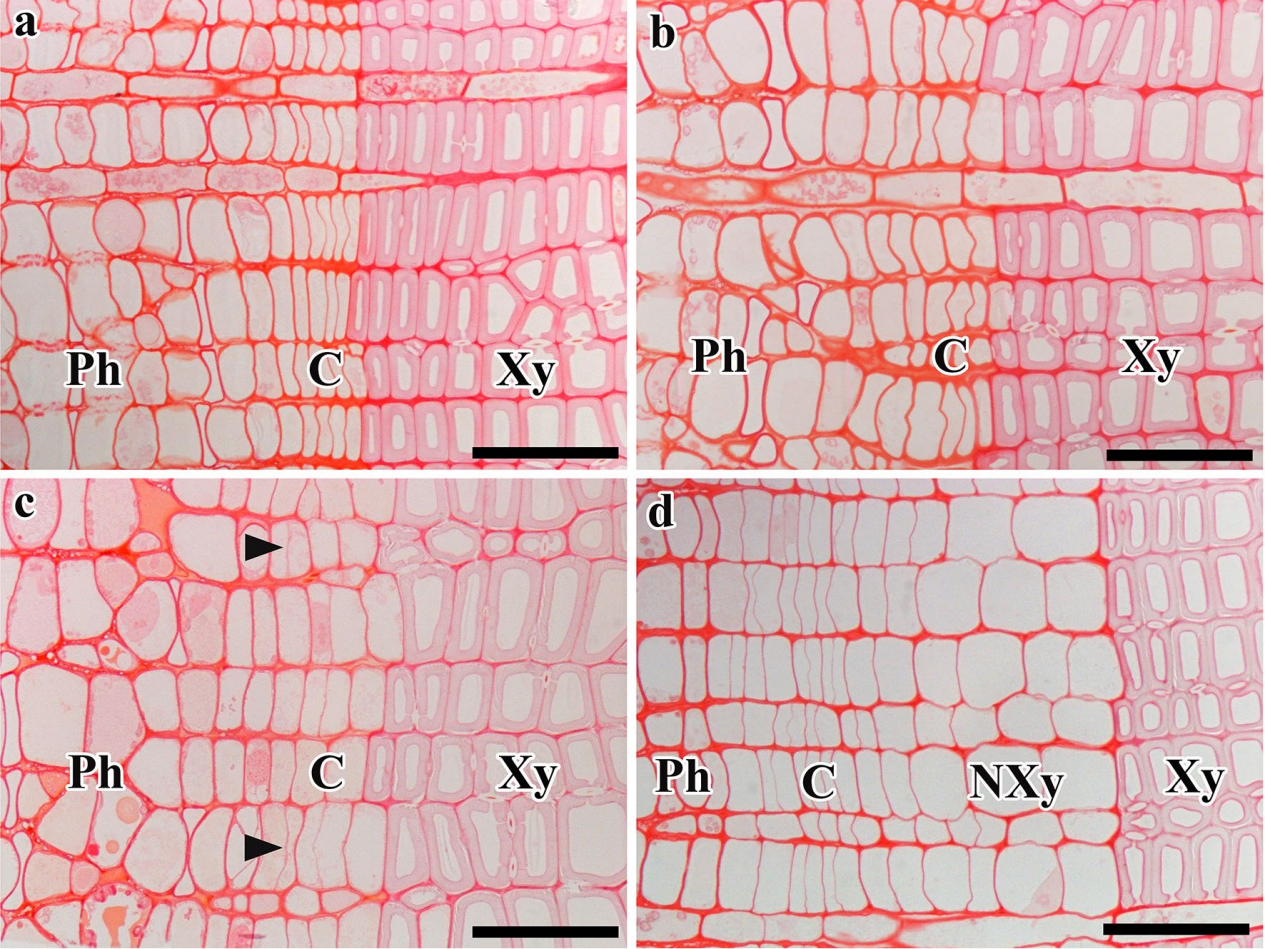
Light micrographs showing transverse views of cambium and its derivatives. In 2015, cambium was dormant on 27 February (**a**) and on 10 March (**b**). On 29 March 2015, new cell plates were observed in the cambium (arrowheads) (**c**). On 9 April 2015, cambial cells had started to differentiate into earlywood tracheids (**d**). *C* Cambium, *Ph* phloem, *Xy* xylem, *Nxy* new xylem. Bars = 50 µm.

In 2015, we performed light microscopic analysis of samples from the two trees that we studied in 2013 and 2014 and from two additional trees, a total of four trees of the same species. Cambium in all four trees was dormant from 27 February to 10 March in 2015 (Fig. 5a,b). Cambial reactivation occurred in the stems of two trees on 25 March 2015 and in stems of the two other trees on 29 March 2015 (Fig. 5c). The formation of earlywood tracheids was initiated on 9 April 2015 in all four trees (Fig. 5d).

### Cambial reactivation at predicted increase of temperatures

**Figure 6:**
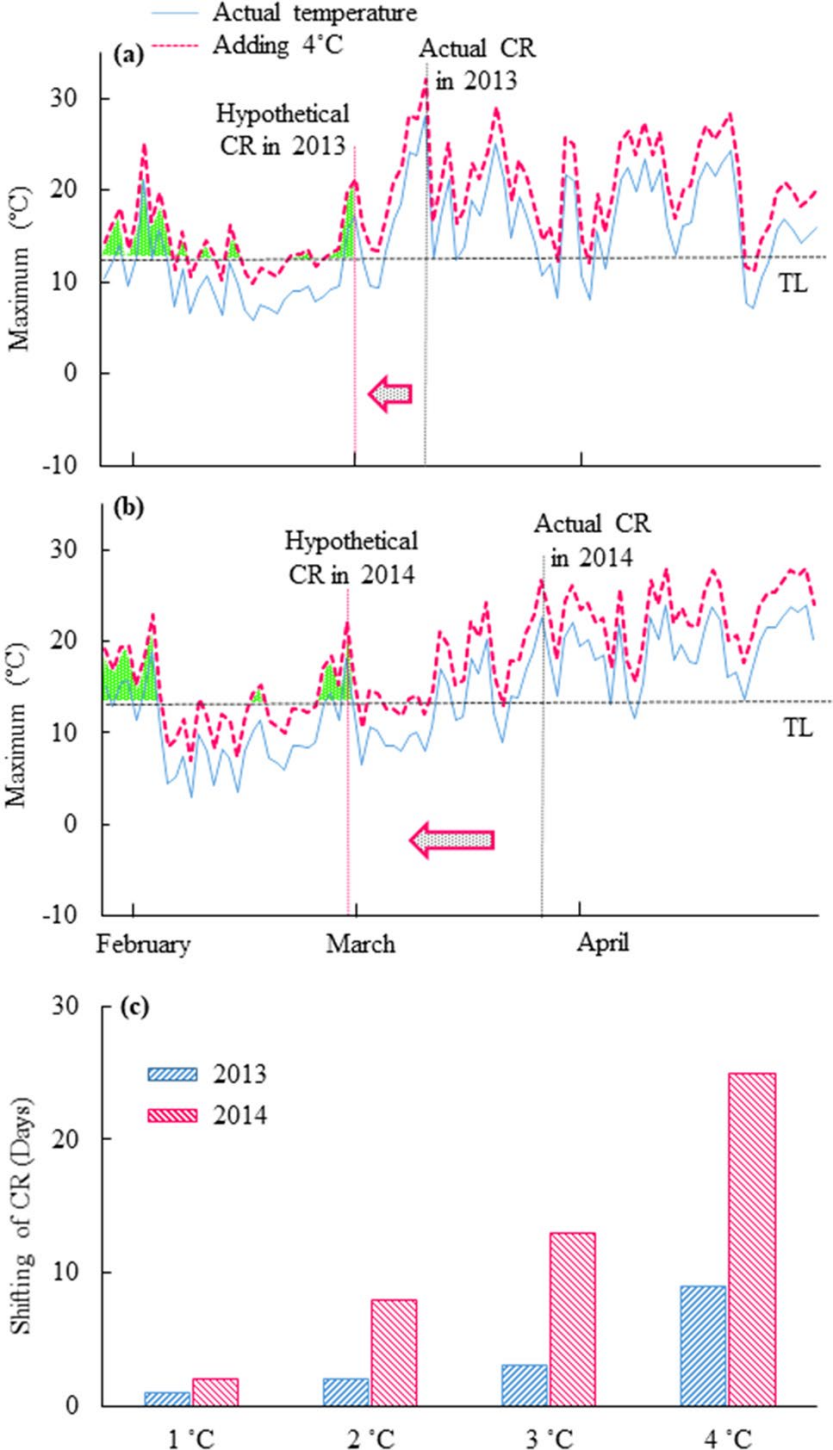
Graphical representation of the CRI after mathematical addition of 4 °C to actual temperatures in 2013 (**a**) and 2014 (**b**). When 4 °C was added mathematically to actual recorded temperatures, the hypothetical timing of cambial reactivation shifted to earlier dates in 2013 (**a**) and 2014 (**b**; red arrows). The horizontal black dotted lines indicate the threshold temperature level (TL) and the vertical black dotted lines indicate cambial reactivation (CR). Green dotted areas indicate the accumulated temperatures above the threshold (**a**,**b**). The changes in the timing of cambial reactivation for the mathematical addition of 1–4 °C to actual temperatures in 2013 and 2014 are presented in (**c**). The horizontal axis shows the mathematical addition in temperatures. The vertical axis shows the difference in days between the hypothetical and actual dates of cambial reactivation.

The timing of cambial reactivation was examined after adding, mathematically, 1–4 °C to the actual temperature profiles of 2013 and 2014 using the CRI. When 1 °C, 2 °C, 3 °C and 4 °C were added to the actual daily maximum temperatures, the calculated timing of cambial reactivation was earlier than the actual date of cambial reactivation in 2013 and 2014. For example, when we added 4 °C mathematically to the actual daily maximum temperatures, the cambial reactivation was estimated to begin on 1 March and 28 February in 2013 and 2014, respectively (Fig. 6a,b). While, at the current conditions, the accumulation of temperatures above 13 °C occurred mainly in March, when 4 °C was added to the daily maximum temperatures, the accumulation of temperatures above 13 °C may occur in January and February (Fig. 6a,b). The calculated timing of cambial reactivation was 9 days and 25 days earlier under this hypothetical condition than under the actual conditions in 2013 and 2014. Our results showed obvious differences in the calculated timing of cambial reactivation between the two consecutive years depending on the predicted increase of daily maximum temperatures (Fig. 6c).

## Discussion

### Effect of temperatures on cambial reactivation in different tree species

We examined the timing of cambial reactivation in response to the sum of daily maximum temperatures (in degrees) in two *Chamaecyparis pisifera* trees under natural conditions in 2013 and 2014. When it is warmer than usual from late winter or early spring, cambial reactivation is initiated earlier in conifers such as *Larix decidua*, *Pinus cembra*, *Picea abies*, *Picea mariana*, *Pinus leucodermis*, *Cryptomeria japonica*^9,37,56,58,59^ and hardwoods such as hybrid poplar (*Populus sieboldii* × *P. grandidentata*)^12^ and *Quercus serrata*^42^. Moreover, an artificial increase in the temperature of the stem during cambial dormancy in winter serves as a direct trigger for the initiation of cambial reactivation in both conifers and hardwoods^6,7,38,39,42^. Therefore, Begum et al.^6,7^ postulated that warmer springs would induce earlier cambial reactivation, which might result in a longer growth period for the cambium of temperate trees. Our results are in line with previous reports that investigated the relationship between winter-spring temperatures and cambial reactivation. The increase in daily maximum temperatures from late winter to early spring started approximately 16 days earlier in 2013 than in 2014, indicating that early spring was warmer in 2013 than in 2014. The timing of cambial reactivation was also 15 days earlier in 2013 than in 2014. These observations suggest that a close relationship might exist between the start of the increase in ambient temperature and the timing of cambial reactivation from late winter to early spring.

We used the CRI to determine the threshold maximum temperature for *C. pisifera* trees, as previously used in the case of deciduous hardwood hybrid poplar^12^ and the evergreen conifer *Cryptomeria japonica*^37^. In hybrid poplar stems, cambial reactivation occurred from 1 April to mid-April in 2005 and 2007 and the threshold maximum temperature was 15°C^12^. In the evergreen conifer *Cryptomeria japonica*, cambial reactivation in stems occurred from late February to mid-March in 2007 and 2008 when the threshold maximum temperature was 10 or 11°C^37^. Similarly, in the present study of the evergreen conifer *C. pisifera*, cambial reactivation was initiated from 10 March to the last week of March in 2013 and 2014 and threshold maximum temperature for cambial reactivation was 13 °C. Our present results show that the timing of cambial reactivation and the threshold maximum temperature for *C. pisifera* stems differed from those of *Cryptomeria japonica,* even though the trees were grown at the same location in Tokyo, Japan. Similarly, localized heating of the stems of *C. pisifera* induced cambial reactivation in winter but heating of longer duration was required for cambial reactivation than in other conifers such as *Cryptomeria japonica*^39^. Thus, it appears that differences among species in the responses of cambium to rising temperatures might be closely related to species-specific sensitivity to temperature of the cambium as it changes from a dormant to an active state. Furthermore, interspecific differences in sensitivity to temperatures might be also important as it has been shown for the phenology of different ecological forms or ages of the same species^20,60^.

Seo et al.^61^ proposed that the accumulation of temperature (in degrees) above a threshold value of 5 °C, expressed in degree-days, could be used to predict the effect of temperature on the date of onset of cambial activity in *Pinus sylvestris* grown in northern Finland. In the present study, cambial reactivation occurred in *C. pisifera* when the threshold maximum temperature was 13 °C and accumulated temperatures were 66 °C and 65 °C in 2013 and 2014, respectively. Therefore, we postulated that certain accumulation of temperature, in degrees, above a specific threshold maximum temperature that is characteristic for the region and species, might be critical in determining the timing of initiation of cambial reactivation.

### Pattern of accumulation of daily temperatures and prediction of cambial reactivation

In Tokyo, Japan, cambial reactivation occurred in hybrid poplar stems when the daily maximum temperature exceeded the threshold maximum temperature 15 °C for 8–10 days, with accumulated temperatures of 93 °C and 96 °C in 2005 and 2007^12^. By contrast, in *Cryptomeria japonica*, cambial reactivation occurred when the daily maximum temperature exceeded threshold maximum temperatures of 10 °C or 11 °C for 25 to 29 days, with accumulated temperatures of 94 °C and 97 °C (for 55-year-old cambium), and 69 °C and 71 °C (80-year-old cambium) in 2007 and 2008, respectively^37^. In the present study, cambial reactivation occurred in *C. pisifera* stems when the daily maximum temperature exceeded a threshold maximum temperature of 13 °C for 11 and 18 days, with accumulated temperatures of 66 °C and 65 °C in 2013 and 2014. The difference between 2013 and 2014 in the time required for achieving accumulated temperatures above the threshold maximum temperature was 7 days. Therefore, we postulated that different patterns of increases in daily temperatures from late winter to early spring dramatically influence the timing of the initiation of cambial reactivation in different years.

In a study of boreal forests in Quebec, Canada, cambial reactivation was estimated to occur three to five days earlier per degree of increase in temperature above threshold minimum (4 °C), average (9.8 °C) and maximum (15.1 °C) temperatures in *Picea mariana* stems^9^. In the present study, we confirmed the validity of the calculated CRI via an examination of the actual date of cambial reactivation in 2015 and daily maximum temperatures from late winter to early spring. When the threshold maximum temperature was taken as 13 °C, the accumulated temperature above this value was calculated as 65 °C on 29 March 2015, suggesting a possible date for cambial reactivation in *C. pisifera* stems. Microscopic analysis revealed that, in the case of two tree stems, cambial reactivation had occurred on 29 March 2015 and, in another two tree stems, it had occurred on 25 March 2015. Thus, the postulated and actual dates of cambial reactivation were similar. Therefore, our results suggest that the CRI allows us to predict the date of onset of cambial reactivation without continuous sampling of tree stems.

### Effect of warmer climates on cambial reactivation and onset of xylem differentiation

We estimated the hypothetical effect on the timing of initiation of cambial reactivation if the temperature had been 1–4 °C higher than the actually recorded daily maximum temperatures in 2013 and 2014. When we added 4 °C, mathematically, to the actual daily maximum temperatures, the hypothetical timing of cambial reactivation shifted from the actual date of 10 March to 1 March in 2013 and from the actual date of 25 March to 28 February in 2014. Therefore, we postulated that, if future temperatures are higher than the actual daily maximum temperatures in 2013 and 2014, the timing of cambial reactivation might be shifted to an earlier date. However, estimates of changes in the timing of cambial reactivation are strongly influenced by the pattern of increases in daily temperatures from late winter to early spring. Furthermore, in the event of higher daily temperatures in Tokyo, the timing of cambial reactivation might be influenced by the pattern of daily temperatures variation in earlier months of the year, such as January and February.

During warm springs, when cambium reactivates earlier, the duration of cambial activity increases leading to annual xylem growth increases in stems of *Picea mariana*^10^. Under the eastern monsoonal climate zone of China, Zheng et al.^62^ observed a close association between winter temperature especially mean temperature of the coldest month and xylem development and structure in several angiosperm species. In a study on the stems of *Pinus sylvestris* trees growing under alpine climatic condition in Austria, it was found that the sum of daily temperatures above the threshold minimum temperature triggers the onset of xylem growth in early spring^63^. From the analysis of tree rings in several species of conifer growing in Tibetan Plateau, Yang et al.^45^ found that an increase of 1 °C in daily minimum temperature of spring advanced the start of xylem production by approximately 6–7 days. The temperatures at different elevations in the spring were closely related to the onset of radial growth of trees with microcore data in *Qilian juniper* growing in Tibetan Plateau^64^, in *Picea abies* and in *Pinus sylvestris* growing in Finland^65,66^ and dendrometer observation in *Larix decidua* growing in Switzerland^50^. They proposed that the temperatures in spring were the most critical factor for the onset of radial growth and the duration of xylem production increased by approximately 7 days for per degree of increase daily temperatures in spring. Rossi et al.^44^ observed that the temperatures in spring produced the best models for predicting the date of xylem growth. They found, in several species of conifers in North America, Europe, and Asia, that the duration of xylem production increased at a rate of approximately 7 days per 1 °C increase of average annual temperatures from − 2 to 12 °C. Prislan et al.^67^ observed a positive correlation of phenological events with temperature in spring and predicted that the duration of the growing season may increase by 20 days over the next six decades, resulting in 38–83% wider xylem increments in *Fagus sylvatica* under the selected climate change scenarios. In the present study, we found that warmer spring induced earlier xylem differentiation as a consequence of earlier cambial reactivation. Moreover, it appeared that the pattern of increases in daily temperatures from winter to spring might be critical for the start of xylem differentiation via its effect on the timing of cambial reactivation. Thus, the pattern of increases in daily temperatures is an important factor that cannot be ignored in estimates of the impact of global warming on the radial growth of trees.

In conclusion, it appears that increases in air temperature from late winter to early spring are very important in the regulation of the timing of cambial reactivation. During the breaking of cambial dormancy, the accumulation of temperatures (degrees) in excess of species-specific threshold temperatures might be critical in determining the timing of cambial reactivation. Moreover, patterns of increases in daily temperatures from late winter to early spring influence the accumulation of temperatures (degrees) above the threshold and also impact the timing of cambial reactivation. The combination of the CRI with meteorological data allows simple and inexpensive estimation of the timing of cambial reactivation without continuous sampling of tree stems. Moreover, the combination of the CRI with predicted warmer temperatures will allow predictions of the timing of cambial reactivation as global climates change.

## Methods

### Plant materials

Two adult specimens of the evergreen conifer *Chamaecyparis pisifera* (age, approximately 50 years; height, approximately 15–18 m; average diameter of stems at breast height, 35 cm) were used in 2013 and 2014. These trees were growing in the field nursery of Tokyo University of Agriculture and Technology in Fuchu, Tokyo, Japan (35.6840° N, 139.4786° E). The adult trees were subjected to sequential observations at the microscopic level of the initiation of cambial reactivation and the differentiation of xylem from late winter to early spring in 2013 and 2014. Samples were collected from the same two trees for two consecutive years.

We identified the species by ourselves using botanical materials. The samples including the permanent histological slides are kept at the laboratory.

In addition, in 2015, we used a total of four adult specimens of *C. pisifera,* namely, the two trees used in 2013 and 2014 plus two additional trees. The age, height and stem diameter at breast height of two additional trees were similar to those of the two trees used in 2013 and 2014. All trees were grown on the same location and samples for microscopy from all four trees were collected in 2015 to evaluate the accuracy of the predicted timing of cambial reactivation that was based on our observations in 2013 and 2014. The main reason for adding two additional trees with two old trees in 2015 is to confirm the hypothesis that CRI model can be applied on the other trees of same species that were similar height and stem diameter and growing on the same location.

### Collection of samples

In 2013 and 2014, samples were collected at 1–5 day intervals from 28 January to 19 April, for 81 days, from both trees. In 2015, four samples from four trees were collected over the same time period.

Sample blocks were cut from main stems at breast height. A series of small blocks that contained phloem, cambium and xylem were removed with a hammer and chisel from each main stem^37–39,68,69^. To eliminate any effects of wounding, samples were removed in a zigzag pattern. No wounding or injury that might have affected tree growth was visible to the naked eye after the collection of each sample from each stem. Blocks were cut into 2-mm-thick sections immediately after removal from trees.

Samples were collected at one-day intervals for the first 3 weeks after the start of sampling in 28 January and then at 3–5 day intervals until the end of the study in April 19. A total of 26 block samples were collected from the stem of each tree in a year.

### Preparation of samples for light microscopy

The samples from blocks of tissues were fixed in 4% glutaraldehyde in 0.1 M phosphate buffer (pH 7.3), under a vacuum, for 1 h at room temperature^37,38,41,68,69^. Fixed samples were washed in 0.1 M phosphate buffer and trimmed to 3 mm in length. After trimming, samples were dehydrated in a graded ethanol series and embedded in epoxy resin (Epon 812 Resin mixed with expoxy hardener Methyl Nadic Anhydride (MNA); TAAB Laboratories Equipment Ltd., UK and DMP-30, Nisshin Em Co., Ltd., Tokyo, Japan). Transverse and radial thin sections were cut from embedded samples at a thickness of approximately 1 µm with a glass knife on an ultramicrotome (Ultracut N; Reichert, Vienna, Austria). These sections were stained with a solution of 1% safranin in water for 30 min and then washed five or six times with water for visualization of cambial cell division and differentiation^37–39,41,68,69^.

All sections were examined under a light microscope (Axioscop; Carl Zeiss, Oberkochen, Germany) as described by Begum et al.^41,68^ and Nakaba et al.^70^.

The reactivation of cambium was determined by the occurrence of periclinal cell walls as visualized by light microscopy^33,38–40,42,69^. To identify the exact timing of the cambial reactivation, we investigated the cambium from several epoxy-embedded 1-µm-thick transverse sections^38,39,41,42,71^ under the light microscope from sequentially collected block samples on each date.

In conifers, more than 90% of fusiform cambial cells in the cambial zone differentiate into xylem cells (tracheids)^72^. Tracheids increase in length by only 5–15% but they expand radially by 200–500% during differentiation. We identified expanding or newly developing xylem cells in the cambial zone by assessing the stage of expansion and by staining of secondary wall of tracheids^7,37–39,68,73^.

We used safranin as staining reagent because it can bind to negatively charged carboxylic groups of non-lignified walls^74,75^. The intensity of safranin staining of cambium is weaker compared to the staining of xylem, particularly after destaining in water or alcohol series. However, we have traditionally used safranin for staining epoxy embedded sections because it penetrates into the resin and allows us to use fluorescence microscopy for observation of both cambium and xylem^7,38,39,41,42,70,76,77^.

### Cambial reactivation index (CRI)

Meteorological data were obtained from the Japan Meteorological Agency in Fuchu, Tokyo. The daily maximum, average and minimum air temperatures from 28 January to 30 April in 2013 and in 2014 are shown in Fig. 3.

The cambial reactivation index (CRI) was defined as the sum of degrees centigrade in excess of a threshold value of the daily maximum temperature from the start of the experiment to the initiation of cambial reactivation, as described by Begum et al.^12,37^. The CRI was calculated as follows:

$${\text{CRI }} =\Sigma \left( {T_{{{\text{md }} - }} T_{{\text{t}}} } \right)$$

where *T*_md_ is the daily maximum temperatures in excess of a given threshold temperature and *T*_t_ is the given threshold temperature. The daily maximum temperatures at the site of the experiment in 2013 and 2014 were used for calculations of CRIs. We tested a range of threshold temperatures from 10 to 14 °C for determination of the CRIs.
